# Cognitive context and impulsivity in adolescence: a comparative study of drama and computer science students using the Barratt Impulsiveness Scale

**DOI:** 10.25122/jml-2025-0116

**Published:** 2025-11

**Authors:** Alina Mihaela Munteanu, Teodor-Cristian Radoi, Monica Petrescu, Cristiana Susana Glavce, Suzana Turcu, Andrei Kozma

**Affiliations:** 1 Medical Anthropology, Francisc I Rainer Institute of Anthropology, Bucharest, Romania; 2 University Politehnica, Bucharest, Romania; 3 Research Department, Alessandrescu-Rusescu National Institute for Mother and Child Health, Bucharest, Romania

**Keywords:** self-control, teenagers, drama, computer science, Barratt impulsiveness scale (BIS)

## Abstract

Impulsivity—acting without forethought, often under emotional pressure—is a prominent adolescent trait, linked to an imbalance in brain maturation. The limbic system, responsible for emotions, matures earlier than the prefrontal cortex, which shapes self-regulation. Educational contexts emphasizing different cognitive demands (emotionally expressive - drama versus analytically focused - computer science disciplines) may influence how impulsivity is expressed. This study examined differences in impulsivity levels among adolescents enrolled in drama and computer science programs. It was hypothesized that drama students would show higher impulsivity due to the spontaneous and emotionally driven nature of their training. Participants included 180 Romanian high school students (ages 14–17) from Dinu Lipatti National College of Arts (drama) and Grigore Moisil National College of Informatics (computer science). Impulsivity was measured using the Barratt Impulsiveness Scale (BIS-11), assessing attentional, motor, and non-planning components. Non-parametric tests were applied due to non-normal data distribution. Drama students showed significantly higher overall impulsivity (Mdn = 75.00) than computer science students (Mdn = 68.75), U = 642.000, *P* < .001, r = –.729). They also displayed greater attentional instability, more motor impulsivity, and lower planning. A moderate to strong inverse relationship between study time and impulsivity was found in the drama group (χ^2^(48) = 83.868, *P* = .003, Cramér’s V = .557), but not in the computer science group. Educational context cognitive demands influence adolescent impulsivity. Emotionally expressive environments like drama may amplify impulsivity, while analytical settings foster greater self-regulation. Findings may guide targeted interventions to support adolescent self-control based on cognitive profiles.

## Introduction

Impulsivity is typically defined as the tendency to act rapidly without prior cognitive deliberation, mainly in response to emotional stimuli. This trait is particularly salient during adolescence, the developmental period marked by increased emotional reactivity and a preference for immediate gratification [1]. Neurobiological findings explain heightened impulsivity by the asynchronous maturation of brain structures: while the limbic system—responsible for emotion and reward processing—is already fully developed by adolescence, the prefrontal cortex, involved in executive control and decision-making, continues to mature into the mid-twenties [2]. This imbalance predisposes adolescents to emotionally driven, impulsive behaviors.

In educational contexts such as drama studies, impulsivity may facilitate creativity through enhancing emotional expressiveness and spontaneity [3]. However, low self-regulation can adversely affect discipline, concentration, and teamwork. In contrast, computer science education emphasizes sustained attention and analytical thinking, where elevated impulsivity may interfere with cognitive persistence and task-focused engagement.

Understanding how impulsivity manifests across domains with distinct cognitive demands is essential for developing targeted interventions. Prior research identifies several contributing factors. Firstly, neurobiological asymmetry — the early maturation of the limbic system alongside delayed prefrontal development — weakens inhibitory control [4]. Secondly, genetic predispositions may interact with environmental influences; specifically, dopamine-related genes have been linked to self-regulation, and contextual factors can amplify impulsive behaviors [5]. Another contributing factor is the family dynamics, which play a relevant role, as adolescents often get the model of impulsivity observed in parents or caregivers [6]. Additionally, socio-cultural contexts influence behavioral patterns: for instance, in traditional societies with greater adolescent autonomy, oppositional behaviors tend to be absent or less frequent [7]. Finally, early attachment styles predict later self-control capacities, with insecure attachments associated with increased emotional reactivity and lower impulse regulation [8].

Despite extensive literature on adolescent impulsivity, few studies have compared this issue across educational domains with divergent cognitive demands, such as drama and computer science. This study addresses this gap, considering the potential implications of impulsivity for academic performance, social functioning, and long-term outcomes.

In the Romanian upper secondary education system, students are distributed across three major tracks: theoretical, technological, and vocational. Drama is part of the vocational–artistic track, with admission based on artistic aptitudes (e.g., expressiveness, creativity, spontaneity), likely attracting students with distinct emotional and cognitive profiles. In contrast, computer science belongs to the theoretical sciences track, with admission based on academic performance in mathematics and informatics, favoring students with high cognitive control. These structural and selection differences may contribute to trait-level differences in impulsivity.

This study used the Barratt Impulsiveness Scale (BIS), a validated tool for measuring attentional, motor, and non-planning dimensions of impulsivity [9]. Initially developed by Ernest Barratt in 1959, the scale has been subjected to multiple adaptations, including versions validated for adolescents in various cross-cultural contexts [10,11]. The working hypothesis posits that drama students would display higher impulsivity scores, while computer science students would exhibit lower levels, consistent with the emotional versus analytical demands of their respective disciplines.

## Material and Methods

### Study design and participants

This study included two independent samples of adolescents aged between 14 and 17 years. The first sample consisted of 90 students (with a 1:1 gender ratio) from the Dinu Lipatti National College of Arts, while the second sample included 90 students from the Grigore Moisil National College of Informatics in Bucharest, Romania (with a gender ratio of approximately 0.95:1). The participants were enrolled in the first year of upper secondary education at the time the study was conducted. Information regarding participants’ psychiatric history or psychological issues was not available, as none of the adolescents were diagnosed with special educational needs (SEN). Descriptive data were collected in accordance with GDPR regulations and ethical standards applicable to research conducted during the COVID-19 pandemic. Throughout this period, the exclusive use of online procedures — combined with heightened sensitivity around collecting personal information, especially in educational settings — increased the likelihood of parental concern or noncompliance. Due to limitations in obtaining sensitive socio-demographic data, only non-identifiable variables — age, sex, area of residence, and family structure — were included. The sample was balanced and tailored to the topic of research, the two groups showing a high degree of similarity in terms of age distribution and sex ratio, with comparable demographic structures regarding area of residence and family background. These characteristics support the suitability of the samples for comparative analysis within the present study (Table 1). Data collection was conducted in October 2021 through an online platform designed for this purpose, where the assessment tool (BIS scale) had been previously uploaded. All participants completed the assessment.

**Table 1:** Descriptive characteristics of the study sample by group (*n* = 180)

Sample	Sex ratio M/F	Age average 14 – 17 years	Area of residence		Family type		
			Urban	Rural	Nuclear	Single-parent	Extended
Drama sample *n* = 90	1: 1	90 (100%)	84 (93.3%)	6 (6.7%)	62 (68.8%)	20 (22.2%)	8 (8.8%)
Computer science sample *n* = 90	0.95: 1	90 (100%)	89 (98.9%)	1 (1.1%)	77 (85.5%)	12 (13.3%)	1 (1.1%)

### Measurement tool

The instrument used for data collection was the Barratt Impulsiveness Scale [9]. It contains 30 items, of which 11 are reverse-scored. The scale is structured into first-order factors (attention, motor, self-control, cognitive complexity, perseverance, cognitive instability) and second-order factors: attentional impulsivity, motor impulsivity, and non-planning impulsivity [12]. A total score of 72 on the BIS is considered indicative of a normal level of impulsivity; scores above this threshold suggest above-average impulsivity levels.

This study focused on the three second-order factors, as they reflect the primary types of impulsivity. All participants completed the questionnaire through individual accounts previously created on the digital platform designed for this purpose. Their responses were recorded and stored securely.

### Data analysis

Data analysis was conducted using IBM SPSS Statistics, version 26 (IBM Corp., Armonk, NY, USA). The overall impulsivity score was calculated by summing all item scores, while subscale scores for attentional, motor, and non-planning impulsivity were calculated separately. To assess the distribution symmetry of the scores, Shapiro–Wilk tests for normality were applied for each set of data. Due to the observed non-normal distribution of the data, non-parametric statistical methods were used. The Mann–Whitney U test was conducted to compare the median overall impulsivity scores and the subscale scores between the two groups. Furthermore, percentile analysis was performed to explore the relative distribution of impulsivity levels across both samples.

To evaluate the relationship between daily individual study time and total BIS scores, a Chi-square (χ^2^) test was applied, and Cramer’s V coefficient was calculated to determine the strength of association between study time and impulsivity levels. Additionally, the effect size was determined to estimate the magnitude of the observed differences between groups. For the non-parametric Mann–Whitney U test, the effect size was calculated using the standardized test statistic (*r = Z / √N*), where *Z* represents the standard score derived from the U distribution and *N* is the total number of participants. Effect sizes were interpreted according to Cohen’s conventions, with *r ≈ 0.10* indicating a small effect, *r ≈ 0.30* a medium effect, and *r ≥ 0.50* a large effect.

## Results

A comparative analysis was conducted on the BIS-11 scores between adolescents studying drama and computer science. The aim was to assess overall impulsivity levels and their specific subdimensions: attentional, motor, and non-planning impulsivity (Table 2).

**Table 2:** Mann–Whitney U test results comparing impulsivity dimensions between drama and computer science adolescents (*n* = 180)

Item	Impulsivity indicator behavior	M_r_drama	M_r_Info	U	Z	*P*	r	Effect size interpretation
1	Non-planning impulsivity	67.00	114.00	2284.500	−5.447	<.001	.406	Moderate
2	Tendency to act impulsively	122.50	58.50	1170.000	−9.552	<.001	.712	Large
3	Spontaneity in decision-making	124.00	57.00	1035.000	−9.990	<.001	.744	Large
6	Inhibition of distracting thoughts	115.38	65.62	1811.000	−7.320	<.001	.545	Large
7	Attention to planning trips	70.88	110.12	2284.500	−5.447	<.001	.406	Moderate
8	Self-control	74.00	107.00	2565.000	−5.954	<.001	.443	Moderate–Large
9	Ability to stay focused	109.91	74.09	2573.000	−4.747	<.001	.353	Moderate
13	Safety-oriented behavior	59.38	121.62	1249.500	−8.936	<.001	.666	Large
15	Preference for complex tasks	80.50	100.50	3150.000	−3.000	<.001	.223	Small–Moderate
16	Change in future projects	101.74	79.26	3038.000	−3.443	<.001	.256	Small–Moderate
23	Concentration on a single task	109.82	71.18	2311.500	−5.803	<.001	.432	Moderate
24	Frequency of changing interests	106.26	74.74	2631.500	−5.004	<.001	.373	Moderate
29	Attraction to puzzle-like tasks	79.93	101.07	3099.000	−2.979	<.001	.222	Small–Moderate
30	Future orientation	78.48	102.52	2968.500	−4.199	<.001	.313	Moderate
Global impulsivity score		128.36	52.64	642.000	−9.782	<.001	.729	Large

Note: Mr = Mean Rank; U = Mann–Whitney U statistic; Z = standard score; *P* = significance level; r = effect size (Cohen’s guidelines: .1 = small, .3 = moderate, .5 = large). Sample sizes: N_drama_= 90, Nc_omputerscience_= 90. Impulsivity was measured using BIS-11.

### Overall impulsivity scores

**Figure 1:**
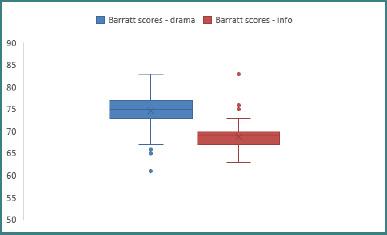
Comparative Barratt scores

The general impulsivity scores for the two samples (Figure 1) were analyzed based on the corresponding percentiles of the weighted averages (Table 3). For the sample studying drama, the scores below the Barratt [12] threshold of normality 72 are concentrated in the 5^th^ and 10^th^ percentiles, while the rest of the results from the 25^th^ to the 95^th^ percentiles correspond to values indicating an impulsivity above the normal average. This distribution implies that most of the sample of adolescents studying drama exhibit a tendency towards impulsive behavior over normal limits. In comparison, the scores of adolescents studying computer science had values below the Barratt threshold of 72 between the 5^th^ and 90^th^ percentiles, indicating that most subjects in this sample exhibit a level of impulsivity within and lower than normal limits.

**Table 3:** Percentiles of the BIS scores for both samples of adolescents

Weighted average	Percentiles						
5	10	25	50	75	90	95
Barratt scores - drama	67.55	71.00	73.00	75.00	77.00	78.00	79.50
Barratt scores - computer sciences	64.59	65.92	66.95	68.75	70.67	72.52	73.21

As the Shapiro–Wilk test indicated non-normal distributions for the Barratt scores in both samples (Table 4), the non-parametric Mann–Whitney rank test was applied.

**Table 4:** Results of the Shapiro–Wilk normality test for the scores in both samples

Shapiro Wilk Test		
Barratt scores	Statistic	Sig. (p)
Drama	.961	.008
Computer science	.945	.001

A Mann–Whitney rank test was conducted to determine if there were statistically significant differences between the mean ranks (M_r_) of the scores obtained by the adolescents studying drama (M_r_drama = 128.36) compared to those studying computer science (M_r_info = 52.64). For the two samples (N_1_ = N_2_ = 90), the result suggested a significant difference between the two groups (U = 642.000, Z = −9.782, *P* < .001), with a large effect size (r = −.729). The findings indicated that the mean ranks for computer science students were significantly lower than those for drama students.

### Attentional impulsivity

**Figure 2:**
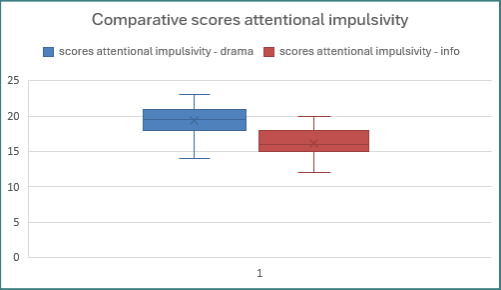
Comparative scores of attentional impulsivity

The analysis of the scores on the attentional impulsivity subscale (Figure 2) was based on its eight constituent items: Item 5, *‘I do not pay attention to what is happening around me’*; Item 6, *‘I have thoughts racing through my head’*; Item 9 (reverse-scored), *‘I can focus easily’*; Item 11, *‘I am restless while reading and playing’*; Item 20 (reverse-scored), *‘I am a calm thinker’*; Item 24, *‘I change my interests frequently’*; Item 26, *‘When I think about something, I often have distracting thoughts’*; and Item 28, *“I am restless at the theatre or cinema”*.

Since the normality Shapiro–Wilk test indicated that all distributions were asymmetrical, we selected the Mann–Whitney rank test for data analysis to identify the statistically significant differences between the mean ranks.

Statistically significant differences were highlighted in the scores for the following items:

*Item 6:* A Mann–Whitney test for N1 = N2 = 90 revealed statistically significant differences in the control of distracting thoughts between the mean ranks of the scores from the drama sample (M_r_drama = 115.38) and the computer science sample (M_r_info = 65.62), U = 1811.000, Z = -7.320, *P* < .001. The effect size was large (r = .545), suggesting that adolescents enrolled in the computer science profile exhibited substantially greater capacity to inhibit distracting thoughts compared to their peers in the drama profile.

*Item 9*: The Mann–Whitney U test highlighted statistically significant differences in the ability to focus on tasks between the mean ranks of the drama adolescents (M_r_drama = 109.91) and the computer science adolescents (M_r_info = 74.09), U = 2573.000, Z = -4.747, *P* < .001, and a moderate effect size (r = .353). The results pointed out that adolescents in the computer science group demonstrated greater efficiency in maintaining task focus compared to their peers in the drama group.

*Item 24*: The Mann–Whitney U test identified statistically significant differences in the frequency of changing interests between the mean ranks of the drama adolescents (M_r_drama = 106.26) and the computer science adolescents (M_r_info = 74.74), U = 2631.500, Z = -5.004, *P* < .001. The effect size (r = .373) implied that drama students tend to explore new interests more frequently, whereas computer science adolescents demonstrated greater stability in their choice of hobbies.

### Motor impulsivity

**Figure 3:**
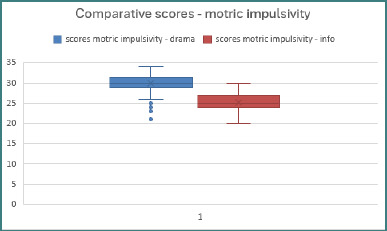
Comparative scores – motric impulsivity

The motor impulsivity subscale consists of 11 items: Item 2, *‘I do things without thinking’*; Item 3, *‘I make snap decisions’*; Item 4, *‘I am interested in immediate fun’*; Item 16, *‘I frequently change my future plans’*; Item 17, *‘I act on impulse’*; Item 19, *‘I act under the influence of the moment’*; Item 21, *‘I frequently change the seat where I sit’*; Item 22, *‘I buy things on impulse’*; Item 23, *‘I can only think about one thing at a time’*; Item 25, *‘I spend more than I have’*; and Item 30 (reverse-scored), *‘I am future-oriented’*. Scores are presented in Figure 3.

As the Shapiro–Wilk normality test indicated a non-normal (asymmetrical) distribution, the non-parametric Mann–Whitney U test was used to determine whether there were statistically significant differences between the mean ranks of the scores obtained by the samples for each item.

Statistically significant differences were found for the following items:

*Item 2*: The Mann–Whitney U test identified significant differences regarding the tendency to do things impulsively between the mean ranks of the scores of adolescents from the drama (M_r_drama = 122.50) and those from computer science (M_r_info = 58.50). The results indicated a propensity for adolescents in drama to react impulsively compared to the computer science sample, U = 1170.000, Z = −9.552, *P* < .001. The large effect size (r = .712) suggested a strong link between educational background and impulsive tendencies, with drama students displaying significantly greater impulsive reactivity than those in computer science.

*Item 3*: The Mann–Whitney U test highlighted statistically significant differences between the mean ranks of the scores of adolescents from the drama (M_r_drama = 124.00) and those from computer science (M_r_info = 57.00). The results suggested a significantly greater spontaneity in decision-making for adolescents in drama compared to those in computer science, U = 1035.000, Z = −9.990, *P* < .001, and a high effect size (r = .744). These findings pointed out both a relevant difference between the groups in this regard and a significant distinction in behavioral tendencies related to interest exploration, as well.

*Item 16*: The Mann–Whitney U test identified statistically significant differences between the mean ranks of the scores of adolescents from the drama (M_r_drama = 101.74) and those from computer science (M_r_info = 79.26). The results indicated that adolescents in drama tend to change their future plans more frequently than computer science adolescents, who showed significantly greater stability in maintaining their intended choices. This behavioral difference was statistically significant (U = 3038.000, Z = −3.443, *P* < .001), with a small-to-moderate effect size (r = .256).

*Item 23*: The Mann–Whitney U test revealed a statistically significant difference between the two groups. Adolescents from the drama class demonstrated higher mean ranks (M_r_drama = 109.82) compared to those from the computer science group (M_r_info = 71.18). The outcomes indicated a greater ability among computer science adolescents to concentrate on a single task, in contrast to drama adolescents, who demonstrated more difficulty focusing. This difference was statistically significant (U = 2311.500, Z = −5.803, *P* < .001), with a moderate effect size (r = .432).

*Item 30:* The Mann–Whitney U test identified a statistically significant difference between the mean ranks of drama students (M_r_drama = 78.48) and the mean ranks of those from computer science (M_r_info = 102.52). These results suggest that computer science adolescents tend to be more future-oriented, whereas drama adolescents appear to focus more on the present. This difference was statistically significant (U = 2968.500, Z = −4.199, *P* < .001), with a moderate effect size (r = .313), indicating a meaningful distinction in cognitive orientation between the two groups.

### Non-planning impulsivity

**Figure 4:**
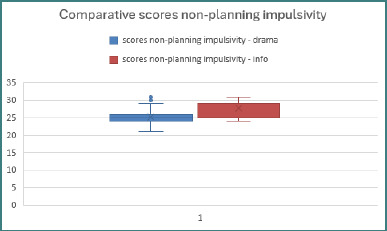
Comparative scores – non-planning impulsivity

The third subscale, non-planning impulsivity (Figure 4), comprises the following 11 items: Item 1 (reverse-scored), *‘I carefully plan my actions’*; Item 7 (reverse-scored), *‘I carefully plan trips in advance’*; Item 8 (reverse-scored), *‘I have good self-control’*; Item 10 (reverse-scored), *‘I save regularly’*; Item 12 (reverse-scored), *‘I am a careful thinker’*; Item 13 (reverse-scored), *‘I think about safety in what I do’*; Item 14, *‘I say things without thinking’*; Item 15, *‘I like thinking about complex problems’*; Item 18, *‘I get bored quickly when solving thinking problems’*; Item 27, *‘I am more interested in the present than in the future’*; and Item 29 (reverse-scored), *‘I like puzzles or similar games’*

First, the Shapiro–Wilk test was applied to assess the normality of data distribution, and the results indicated that the distributions were asymmetrical. Consequently, the non-parametric Mann–Whitney U test was used to determine whether there were statistically significant differences between the mean ranks of scores for each item across the two groups.

*Item 1*: The Mann–Whitney U test revealed a statistically significant difference between drama students (M_r_drama = 67) and those from computer science (M_r_info = 114), indicating higher non-planning impulsivity among adolescents from the computer science group (U = 2284.500, Z = −5.447, *P* < .001). The effect size was moderate (r = .406), suggesting that the difference in non-planning impulsivity between the two groups is both statistically significant and practically meaningful.

*Item 7*: The Mann–Whitney U test marked statistically significant differences between the mean ranks of the scores of adolescents from drama (M_r_drama = 70.88) and those from computer science (M_r_info = 110.12). The statistics indicated that computer science students pay more attention to planning trips than drama students (U = 2284.500, Z = −5.447, *P* < .001), with a moderate effect size (r = .406).

*Item 8*: The Mann–Whitney U test revealed statistically significant differences between the mean ranks of drama students (M_r_drama = 74.00) and computer science students (M_r_info = 107.00). This indicates better self-control among adolescents in the computer science group (U = 2565.000, Z = −5.954, *P* < .001). The effect size was moderate to large (r = .443), showing that the difference in self-control between the two groups is not only statistically significant but also practically meaningful.

*Item 13*: The Mann–Whitney U test revealed statistically significant differences between drama students (M_r_drama = 59.38) and computer science students (M_r_info = 121.62). The results suggest that adolescents in the computer science group are more inclined toward advanced preparation to ensure safety, whereas those from the drama group tend to react more impulsively (U = 1249.500, Z = −8.936, *P* < .001). The effect size was large (r = .666), indicating a substantial and meaningful difference in safety-related preparatory behaviors between the two groups.

*Item 15*: The Mann–Whitney U test revealed statistically significant differences between the drama group (M_r_drama = 80.50) and the computer science group (M_r_info = 100.50). The findings suggest a modest preference for complex tasks among computer science adolescents compared to those from drama (U = 3150.000, Z = −3.000, p < .001), with a small effect size (r = .223).

*Item 29:* The Mann–Whitney U test revealed a significant difference between the drama group (M_r_drama = 79.93) and the computer science group (M_r_info = 101.07). The results indicate that computer science students showed a stronger preference for puzzle-like tasks compared to drama students (U = 3099.000, Z = −2.979, p < .001), although the effect size was small (r = .222).

The analysis of the impulsivity subscale items highlighted statistically significant differences between the two samples, which can be attributed to several factors. Firstly, the higher impulsivity observed in drama students should not be interpreted as a direct consequence of their chosen activity. Rather, it may reflect an interaction between the expressive nature of drama training and the personality traits that predispose individuals to select such an educational profile. Thus, while drama students are encouraged to express their personality spontaneously, be expressive, and live in the moment, the computer science adolescents are focused on logical tasks that are rigorously structured and spread over extended daily time units. By performing different types of activities and emphasizing different ways of approaching tasks, adolescents in the two samples seem to develop different attitudes and reactivity patterns to new situations. While drama students are predisposed to testing new things and paying attention to living the emotion of the moment, computer science adolescents tend to reflect more before reacting to stimuli, regardless of their emotional nature.

Another factor that may contribute to differences in impulsivity between the two groups is the amount of time adolescents spend daily on cognitive tasks (Table 5). A Chi-square test of association between daily study duration and overall Barratt scores revealed a moderate to strong relationship among drama students (χ^2^(48) = 83.868, *P* = .003, Cramer's V = .557). This suggests that the more time drama students devoted to studying, the lower their impulsivity levels tended to be. In contrast, for computer science students, the association between study time and impulsivity was not statistically significant (χ^2^(48) = 50.009, *P* = .394), indicating that other factors may play a more important role in shaping impulsivity within this group.

**Table 5:** Distribution of daily study time among adolescents in drama and computer science classes

	Daily study time (in hours)							Total
1 h	2 h	3 h	4 h	5 h	6 h	7 h	*N*
Adolescents from drama classes	36	42	7	6	-	-	-	90
Adolescents from computer science classes	-	-	3	6	32	47	2	90

## Discussion

This study compared impulsivity profiles in two adolescent groups, performing arts (drama) students and technical education (computer science) students, using the BIS-11. Significant differences were found not only in overall impulsivity but also across the attentional, motor, and non-planning subscales. As anticipated, drama students showed higher impulsivity scores across most percentiles, exceeding the BIS normative threshold from the 25^th^ percentile onward. These findings suggest that educational contexts that emphasize spontaneity, emotional expressiveness, and present-focused engagement, characteristic of drama training, may foster or align with heightened impulsive tendencies. Conversely, computer science students demonstrated lower overall impulsivity, consistent with the structured, problem-solving, and future-oriented nature of their curriculum.

Analysis of the attentional impulsivity subscale revealed that drama students were more prone to intrusive thoughts, distractibility, and rapid shifts in interest. This may reflect a cognitive style receptive to external stimuli and creative exploration inherent in the performing arts. In contrast, computer science students exhibited superior attentional control and sustained focus, aligning with the demands of technical subjects that require logical processing and sequential task completion.

Similarly, motor impulsivity differed significantly between groups. Drama students showed a greater tendency to act without reflection, make snap decisions, and frequently alter plans-behaviors that are often normalized or even rewarded in creative contexts. Computer science students displayed more restraint and future-oriented thinking, as evidenced by higher scores on reverse-coded items related to planning and foresight. The non-planning impulsivity subscale further highlighted these contrasts: computer science students demonstrated stronger planning behavior, heightened safety awareness, and a preference for cognitively challenging tasks, whereas drama students favored present-focused, reactive decision-making. These patterns likely mirror the distinct cognitive and emotional demands embedded within their respective educational settings.

These findings are consistent with Reynolds *et al*. [13], who highlighted the multidimensional structure of impulsivity in adolescents, comprising attentional, motor, and non-planning components. Similar to their conclusions, our results suggest that impulsivity profiles reflect underlying cognitive control mechanisms shaped by environmental influences. The distinct patterns observed between drama and computer science students further support the notion that educational tracks function as cognitive conditioning environments. The nature of daily activities, planning demands, and goal-setting strategies embedded within each curriculum appears to contribute to the development and expression of impulsivity traits during adolescence.

Furthermore, the use of the BIS-11 in this study aligns with Waegeman *et al*.’s psychometric evaluation of cognitive self-regulation [14], which demonstrated that self-report questionnaires capture subjective dimensions of impulsivity and cognitive control that may diverge from behavioral task performance. This distinction is particularly pertinent given the contrasting educational contexts studied here, suggesting that self-perceived impulsivity is shaped by the cognitive and emotional demands specific to these environments. Therefore, while self-report tools like the BIS-11 provide valuable insights into adolescents’ impulsivity patterns, complementing these measures with behavioral assessments would offer a more comprehensive understanding of the multifaceted nature of impulsivity.

To sum up, this study contributes to the growing evidence that impulsivity during adolescence is a complex, multidimensional construct heavily influenced by the context of academic and cognitive experiences. Educational programs not only impart knowledge but also function as environments that shape cognitive traits such as impulsivity, attentional control, and planning strategies.

### Limitations

This study is subject to several limitations. Its cross-sectional design precludes causal inference regarding the relationship between educational specialization and impulsivity. The use of a self-report instrument (BIS-11) may have introduced response biases or not fully captured behavioral manifestations of impulsivity. Additionally, the sample was limited to two institutions in a single urban area; thus, the results cannot be generalized.

### Recommendations for future research

To deepen the insights generated by this study, future research should adopt longitudinal designs to determine whether—and to what extent—academic training actively shapes impulsivity over time. Such an approach would make it possible to trace causal pathways and developmental trajectories rather than relying solely on cross-sectional comparisons.

Additionally, integrating behavioral assessments (e.g., Go/No-Go tasks, delay discounting paradigms) alongside self-report measures would allow for a more comprehensive evaluation of impulsivity by capturing both subjective and objective components. Combining psychometric and neurocognitive data could yield more precise profiles of impulsivity across educational contexts.

Expanding the sample to include multiple schools, both urban and rural, and across different cultural contexts, would also enhance the external validity of the findings. Moreover, including additional academic specializations (e.g., humanities, mathematics, or sports) could further elucidate the relationship between cognitive training and impulsivity profiles.

Finally, future research would examine moderating variables, such as parental education, digital media use, or stress levels, which may influence impulsivity and its expression in academic settings. Experimental interventions, such as the introduction of executive function training in drama programs or creative expression modules in technical curricula, may also provide valuable insights into the plasticity of impulsivity during adolescence.

## Conclusion

This study highlights meaningful differences in impulsivity profiles between adolescents enrolled in drama and computer science educational tracks. Drama students were found to be significantly more impulsive across total and subscale scores, with a higher frequency of behaviors associated with attentional and motor impulsivity. In contrast, computer science students demonstrated greater cognitive control, planning ability, and attentional stability.

These results suggest that the educational environment and the cognitive demands it imposes may influence the development or reinforcement of certain personality traits, including impulsivity. While the expressive nature of drama may encourage creativity and spontaneity, the structured problem-solving approach familiar to computer science education may cultivate cognitive restraint and forward planning. Although participants were in the initial stage of upper secondary education (October 2021) and thus had minimal exposure to their respective curricula, it is plausible that pre-existing psychological predispositions played a role in both their self-selection and admission into the respective educational tracks. Further research is warranted to explore the longitudinal impact of educational discipline on personality development, and whether interventions or cross-training (e.g., introducing planning tasks in drama or expressive tasks in technical education) can moderate these impulsivity tendencies in adolescents.
